# Unblocking Oxygen Charge Compensation for Stabilized High‐Voltage Structure in P2‐Type Sodium‐Ion Cathode

**DOI:** 10.1002/advs.202200498

**Published:** 2022-03-28

**Authors:** He Zhu, Zhenpeng Yao, Hekang Zhu, Yalan Huang, Jian Zhang, Cheng Chao Li, Kamila M. Wiaderek, Yang Ren, Cheng‐Jun Sun, Hua Zhou, Longlong Fan, Yanan Chen, Hui Xia, Lin Gu, Si Lan, Qi Liu

**Affiliations:** ^1^ Department of Physics City University of Hong Kong Hong Kong 999077 P. R. China; ^2^ Center of Hydrogen Science Shanghai Jiao Tong University Shanghai 200240 P. R. China; ^3^ Innovation Center for Future Materials Zhangjiang Institute for Advanced Study Shanghai Jiao Tong University Shanghai 201203 P. R. China; ^4^ The State Key Laboratory of Metal Matrix Composites School of Materials Science and Engineering Shanghai Jiao Tong University Shanghai 200240 P. R. China; ^5^ Department of Chemistry and Chemical Biology Harvard University Cambridge MA 02138 USA; ^6^ School of Chemical Engineering and Light Industry Guangdong University of Technology Guangzhou 510006 P. R. China; ^7^ X‐Ray Science Division Argonne National Laboratory Argonne IL 60439 USA; ^8^ College of Physics and Materials Science Tianjin Normal University Tianjin 300387 P. R. China; ^9^ School of Materials Science and Engineering Tianjin University Tianjin 300072 P. R. China; ^10^ School of Material Science and Engineering Nanjing University of Science and Technology Nanjing 210094 P. R. China; ^11^ Institute of Physics Chinese Academy of Sciences Beijing 100190 P. R. China; ^12^ Center for Neutron Scattering City University of Hong Kong Hong Kong 999077 P. R. China; ^13^ Shenzhen Research Institute City University of Hong Kong Shenzhen 518057 P. R. China; ^14^ Hong Kong Institute for Clean Energy City University of Hong Kong Hong Kong 999077 P. R. China

**Keywords:** high‐voltage structural stability, in situ synchrotron characterizations, layered transition‐metal oxide cathodes, oxygen charge compensation, sodium‐ion battery

## Abstract

Layered transition‐metal (TM) oxides are ideal hosts for Li^+^ charge carriers largely due to the occurrence of oxygen charge compensation that stabilizes the layered structure at high voltage. Hence, enabling charge compensation in sodium layered oxides is a fascinating task for extending the cycle life of sodium‐ion batteries. Herein a Ti/Mg co‐doping strategy for a model P2‐Na_2/3_Ni_1/3_Mn_2/3_O_2_ cathode material is put forward to activate charge compensation through highly hybridized O_2_ *_p_*—TM_3_ *_d_* covalent bonds. In this way, the interlayer O—O electrostatic repulsion is weakened upon deeply charging, which strongly affects the systematic total energy that transforms the striking P2–O2 interlayer contraction into a moderate solid‐solution‐type evolution. Accordingly, the cycling stability of the codoped cathode material is improved superiorly over the pristine sample. This study starts a perspective way of optimizing the sodium layered cathodes by rational structural design coupling electrochemical reactions, which can be extended to widespread battery researches.

## Graphical abstract

Here a Ti/Mg codoping strategy for a model P2‐Na_2/3_Ni_1/3_Mn_2/3_O_2_ (NNM) cathode material is proposed to activate oxygen charge compensation at high voltage. In this way, the interlayer O—O electrostatic repulsion is released, which modifies the P2–O2 transition into a moderate solid‐solution type evolution, and therefore remarkably enhances the cycling performance of the P2‐type NNM layered cathode.

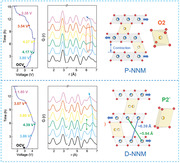

## 1 Introduction

With the growth of the economy, the global demands for energy have significantly increased. However, nonrenewable fossil fuels are being exhausted rapidly, and their over‐exploitation and consumption also lead to severe environmental issues. As a result, the development of energy storage techniques for sustainable energy sources (e.g., solar, wind, etc.) becomes necessary to a green and recycling society.^[^ ^1^ ^]^ Among the currently existing energy storage technologies, lithium‐ion batteries (LIBs) are widely applied in portable devices and preferred for powering next‐generation electric vehicles (EVs).^[^ ^2^, ^3^, ^4^ ^]^ Despite almost 30 years of commercial success, nevertheless, concerns have recently arisen about the shortage of Li resource and potentially rising costs.^[^ ^5^, ^6^ ^]^ With this regard, searching for alternative energy storage chemistries lies at the very heart of global concerns nowadays. Contrary to the lithium raw materials, sodium resources are earth‐abundant, distributed worldwide and cost effective.^[^ ^7^ ^]^ In light of this, the development of sodium‐ion batteries (SIBs), using sodium instead of lithium as charge carriers, shows great promises upon overcoming the economic barriers of LIBs and therefore have attracted significant attention.^[^ ^8^ ^]^

Apart from the prospect, there are still critical challenges for the widespread usage of SIBs. Especially, the exploration of suitable cathode materials has been the focus of attention, since it is the cathode materials that account for most of the overall costs of the whole cell and determine critical battery performance, such as energy density and cut‐off voltage.^[^ ^9^, ^10^ ^]^ In analogy to Li‐based layered cathodes that have been widely applied in commercial LIBs, a variety of sodium layered intercalated compounds, for example, sodium layered transition‐metal (TM) oxides (i.e., Na*_x_* TMO_2_, TM = Ni, Mn, Fe, Cr, V, Ti, etc.), have been extensively explored as cathode materials for SIBs.^[^ ^11^, ^12^, ^13^ ^]^ These materials share common advantages over lithium layered counterparts (e.g., LiCoO_2_), such as high energy density, fast ionic diffusion, and good stability in air,^[^ ^14^, ^15^, ^16^ ^]^ but most of them suffer from severe capacity decay and poor structural stability of Na‐intercalated lattice.^[^ ^17^ ^]^ To improve the cycling stability, substantial efforts with various degree of success have been made including incorporating foreign dopants,^[^ ^18^, ^19^ ^]^ coating protective layers,^[^ ^20^, ^21^, ^22^ ^]^ morphology control,^[^ ^23^, ^24^ ^]^ etc. Nevertheless, more solutions with artful and in‐depth concepts for fine material design are still urgently desired.

**Figure 1:**
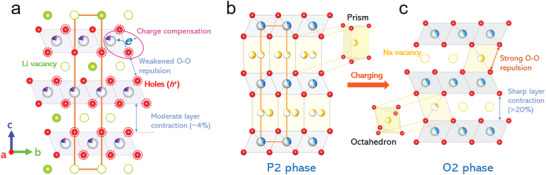
a) The oxygen charge compensation behavior of the Li layered oxides when deeply charged. b) The typical P2 and c) O2 structure emphasizing the Na—O local structures.

The gap between the cycling stabilities of Li‐ and Na‐based layered oxides could be mostly attributed to their diverse structural changes upon Li‐ or Na‐(de)intercalation. Starting from a Li‐intercalated layered structure (**Figure** **1**a), the framework exhibits a continuously evolved interlayer contraction when deeply charged, which is sustainable for long‐term cycling.^[^ ^25^, ^26^ ^]^ A charge compensation mechanism between highly hybridized TM‐3*d* and O‐2*p* states has been thought to be responsible for this moderate solid solution behavior.^[^ ^27^, ^28^ ^]^ In detail, when most of the lithium ions are extracted from the lattice at high voltage, the interlayer distance is governed heavily by the O–O electrostatic force between the layers. Meanwhile, the redox charge loss of TMs could be compensated persistently by the O_2_ *_p_* electrons through generating electron holes (i.e., oxygen charge compensation). This will weaken the O–O repulsion and thereby give rise to moderate interlayer contraction for endurable cycle life. For the sodium layered oxides, the most prevailing structures applied for SIB cathodes are P2 or O3 phase, where Na^+^ ions are accommodated at “prismatic” or “octahedral” sites, and the stacking sequences of oxygen are “ABBA” for P2 and ABCABC for O3, respectively.^[^ ^29^ ^]^ For the O2 cathodes, typically, a phase transition from P2 to O2 occurs upon charging to ≈4.1–4.2 V, accompanied with a striking interlayer shrinkage in distance (≈20%, Figure 1c).^[^ ^30^ ^]^ The lattice mismatch between P2 and O2 results in significant internal stress and mechanical fractures, hence giving rise to poor cycling stability.^[^ ^6^ ^]^ Theoretical studies have indicated that the high‐voltage P2–O2 transition is driven by elevated systematic energy.^[^ ^31^ ^]^ Unlike their Li counterparts with weakened O–O repulsion induced by oxygen charge compensation, the O–O repulsion in Na*_x_* TMO_2_ becomes increasingly stronger along with Na^+^ extraction. This intrinsically increases the systematic total energy, whereby the P2–O2 transition is triggered when overcoming the critical kinetic barrier. Given the above, it is the oxygen charge compensation that acts as a key enabler toward sustainable cycling stability. Hence, an impetus for activating charge compensation in sodium layered cathodes is motivated to relax the strong O–O repulsion at high voltage, and ultimately stabilize the P2 structure for improved cycling performance.

Herein we put forward a synergetic Ti/Mg codoping strategy to realize this scenario, for which the oxygen charge compensation is activated in P2‐type Na_2/3_Ni_1/3_Mn_2/3_O_2_ (NNM), a model system of sodium layered TM oxide,^[^ ^32^ ^]^ for improved cycling stability. Compared with the above‐mentioned optimizing approaches, the doping strategy has been proven to be effective and fundamental, as it is capable of directly tuning the material properties at atomic and electronic levels. It has been found that the Ti/Mg codoped NNM material (Na_2/3_Ni_0.25_Mg_0.083_Mn_0.55_Ti_0.117_O_2_, D‐NNM) shows an endurable cycling performance far superior to the pristine NNM (P‐NNM). In situ X‐ray diffraction (XRD) reveals that the D‐NNM exhibits a moderate solid‐solution‐type behavior at high voltage, instead of the P2–O2 phase transition with striking interlayer contraction observed in P‐NNM. By in situ pair distribution function (PDF) analysis, detailed local structural dynamics have been clearly encoded. Instead of the typical O2 structure, an orthorhombic phase (denoted as P2’ phase) is suggested in the high‐voltage D‐NNM, and this P2’ phase can be transformed continuously from P2 by sliding slabs along (1/3, 1/3, 0) direction. The thermodynamically preferred P2–P2’ transition is also validated with first‐principles calculations. Near‐ambient pressure X‐ray photoelectron spectroscopy (NAP‐XPS) has demonstrated that a charge compensation behavior from O to TMs occurs concomitant with the P2–P2’ transition. The present study initiates a prospective way of stabilizing the P2 structure through activating oxygen charge compensation to develop sustainable sodium layered cathode materials.

## 2 Main Text

**Figure 2:**
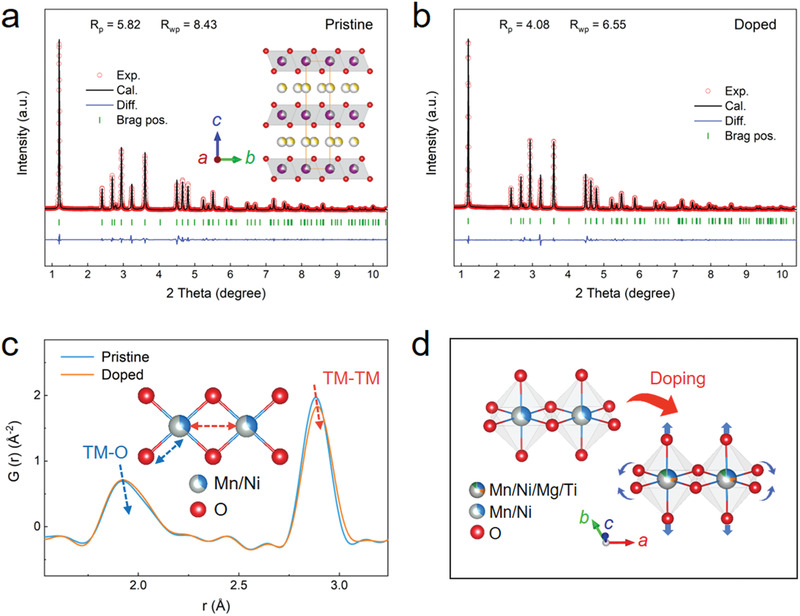
a) Rietveld refinements of the XRD patterns for P‐NNM and b) D‐NNM samples. The inset in a) provides the schematic diagram of the *P*6_3_/*mmc* unit cell used for the refinements. c) The comparison of low‐*r* PDF patterns between P‐NNM and D‐NNM samples. The inset shows the octahedral bonding environment in the P2 structure. d) The schematic diagram of the local octahedral distortion upon doping extracted from the PDF refinements.

The P2‐type P‐NNM and D‐NNM samples were prepared using a high‐temperature solid‐state reaction method (see the Methods in the Supporting Information). From the scanning electron microscopy (SEM) images (Figure S1a,b, Supporting Information), the as‐prepared P‐NNM and D‐NNM samples show a similar brick‐like morphology with particle sizes ranging from 1 to 10 µm. The D‐NNM particles also exhibit uniform distributions of Ti and Mg dopants, as captured by the mappings of energy dispersive spectroscopy (EDS, Figure S1c–h, Supporting Information). The crystal structures of the as‐prepared materials were probed with high‐energy synchrotron XRD (**Figure** **2**a,b). The diffraction peaks of both samples could be well indexed to the hexagonal P2 layered structure (space group: *P*6_3_/*mmc*) without any impurity. Based on the results of Rietveld refinements (Tables S1 and S2, Supporting Information), the D‐NNM shows an expanded *a*‐ and *c*‐axis with the introduction of Mg and Ti dopants. In this codoping system, the Mg and Ti cations are revealed to separately occupy the Ni and Mn sites, respectively, which is consistent with the individually Mg‐ and Ti‐substituted NNMs.^[^ ^17^, ^18^ ^]^ As a result, the lattice expansion of the D‐NNM sample is attributed to the larger ionic radius of Mg^2+^ (0.72 Å) than Ni^2+^ (0.69 Å), as well as that of Ti^4+^ (0.61 Å) than Mn^4+^ (0.53 Å).

The typical P2‐type structure possesses edge‐sharing octahedral chains perpendicular to the *c* direction. Doping Mg and Ti ions into the D‐NNM lattice could induce local octahedral distortion that deviates from the P‐NNM, which has been investigated by synchrotron X‐ray PDF analysis. In the low‐*r* region of the PDF patterns (Figure 2c), the first peak emerging at ≈1.95 Å corresponds to the TM—O bond length, while the second peak at ≈2.90 Å is related to the distance of the nearest TM…TM atom pairs. Compared to the P‐NNM, these two peaks of the D‐NNM shift rigidly to the high‐*r* direction. This result suggests that the volume of the octahedra in the D‐NNM increases with the introduction of dopants, which agrees well with the XRD results. For a further insight into the local bonding geometry, full‐profile refinements of the PDF G(*r*) patterns were carried out with the hexagonal *P*6_3_/*mmc* model (Figure S2, Supporting Information). It is revealed that the octahedra in the D‐NNM show a slight distortion. Upon doping, the TM—O bond length expands from 1.950 to 1.957 Å, while the O—TM—O bond angle opening along the octahedral chain contracts from 84.81° to 84.74° (Figure 2d). Details of the structural information extracted from the PDF refinements are given in Tables S3 and S4 (Supporting Information).

For a further step, the as‐prepared P‐NNM and D‐NNM powders were also examined by a combined X‐ray absorption near edge structure (XANES) and extended X‐ray absorption fine‐structure (EXAFS) analysis of Ni and Mn K‐edges. From the XANES data (Figure S3a,b, Supporting Information), the K‐edge of Ni shifts to a lower energy upon doping, while that of Mn shows no obvious energy shift. Thus, the dopants in the D‐NNM structure give rise to a slight reduction of Ni ions. On the other hand, the real‐space transformed EXAFS data shows that the first two shells of both Ni and Mn expand upon doping (Figure S3c,d, Supporting Information). This suggests an enlarged octahedra in the D‐NNM, which is consistent with the PDF results. The above structural characterizations indicate that our Mg/Ti codoping strategy does not significantly change the P2 structure of the as‐prepared NNMs. Only slight distortions are expected in the D‐NNM sample.

**Figure 3:**
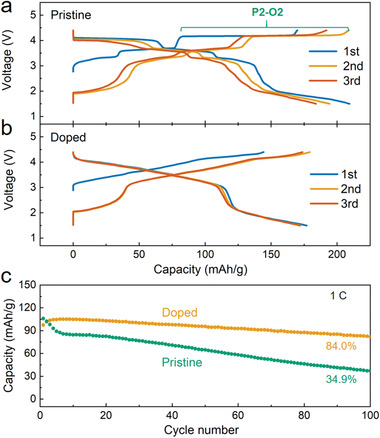
a) The charge–discharge voltage profiles of the P‐NNM and b) the D‐NNM materials for the first three cycles at 0.1 C. c) The comparison of capacity retentions at 1.0 C for 100 cycles.

The electrochemical performance of the P‐NNM and D‐NNM electrodes was evaluated by the coin‐type half cells over the voltage range from 1.5 to 4.4 V. **Figure** **3**a,b shows the voltage profiles of the initial three cycles at a current of 0.1 C (1 C = 170 mAh g^−1^). For the P‐NNM, a series of voltage plateaus could be observed upon charging and discharging (Figure 3a). Among them, the plateaus at ≈3.29/3.21 and ≈3.69/3.62 V are related to the transitions between different Na^+^/vacancy ordering, while the long plateau at ≈4.19/4.02 V could be identified as a biphasic P2–O2 region.^[^ ^33^ ^]^ The first discharge capacity of the P‐NNM reaches a high value of ≈210 mAh g^−1^, attributed from multistep reductions of Ni^4+/3+^/Ni^2+^ (> 2.0 V) and Mn^4+^/Mn^3+^ (< 2.0 V) redox couples.^[^ ^34^ ^]^ In contrast, only Ni^2+^ ions are oxidized during the first charging process, accounting for a lower charge capacity of ≈170 mAh g^−1^. Upon further cycling, the capacity derived from the P2–O2 plateau drops severely with a remarkable increase in polarization. This capacity decay is related to the structural damage induced by repetitive strains during the P2–O2 transition.

For the D‐NNM sample, the plateaus at the cut‐off voltage lower than 4.0 V on the charging‐discharging curves are greatly eliminated, indicating a suppressed Na^+^/vacancy ordering (Figure 3b). It has been established that the superstructure formed by the Na^+^/vacancy ordering impedes the Na^+^ transport, hence detrimental to the electrochemical performance.^[^ ^35^ ^]^ The introduction of foreign dopants is suggested to break the charge ordering of cations and thereby prevent the Na^+^/vacancy ordering.^[^ ^36^ ^]^ At higher voltage (>4.0 V), an uphill plateau is observed for the D‐NNM cathode, in contract to the flat plateau of P2–O2 transition in the P‐NNM. This indicates a modified high‐voltage structural dynamical behavior in the codoped sample. In addition, the high‐voltage capacity decay and the related polarization observed in the P‐NNM are less obvious in the D‐NNM, indicating that the dopants could serve as structural stabilizers in the codoping system. Similar to the P‐NNM, the first discharge capacity delivered in the D‐NNM (≈177 mAh g^−1^) is higher than the first charge capacity (≈145 mAh g^−1^), which is derived from Mn^4+^/Mn^3+^ multiredox couples upon discharge. Nevertheless, the capacity retention of these two materials shows clear distinction (Figure 3c): while the capacity of P‐NNM decays rapidly to 34.9% over 100 cycles, the capacity of D‐NNM is well retained with minimum loss (95.6% for 50 cycles and 84.0% for 100 cycles). These observations suggest a moderate structural evolution in the D‐NNM upon charging–discharging cycles, which accounts for the optimized cycling stability.

**Figure 4:**
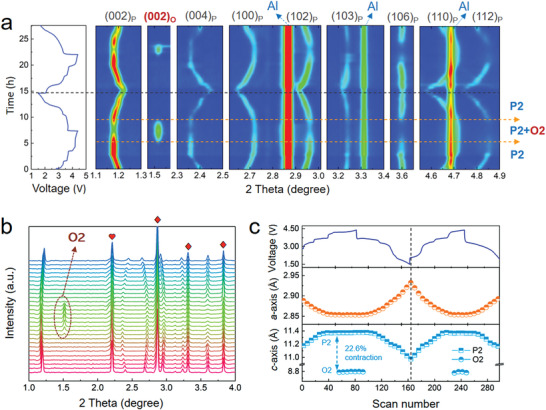
a) Contour plots of the in situ XRD patterns for P‐NNM cell along with their corresponding voltage profiles. b) The stacking profile of the in situ XRD patterns collected during the initial cycle. The brown dashed circle and arrow indicate the newly emerged (002) peak of the O2 phase. The peak marked with heart belongs to the Li‐metal counter electrode, while those marked with rhombuses are generated from Al current collector. c) The lattice parameters of *a*‐axis (upper) and *c*‐axis (lower) extracted from fitting the in situ XRD patterns.

To elucidate the doping impact on the structural stability, in situ synchrotron XRD measurements were performed on both P‐NNM and D‐NNM cells. From the contour plots of the P‐NNM (**Figure** **4**a), the peaks belonging to the P2 phase continuously evolve upon charging and discharging. When the voltage reaches ≈4.2 V, a new set of characteristics emerges at the O2 (space group: *P*6_3_ *mc*) Bragg positions, accompanied with a slight weakening of the P2 intensities. This means a portion of P2 phase transforms to O2 phase, and these two phases coexists in the P‐NNM electrode. The stacking profile of the in situ XRD patterns has been also plotted to detail this behavior (Figure 4b). Upon discharging to ≈3.9 V, the diffraction peaks corresponding to the O2 phase disappear, indicating that the P2–O2 transition is reversible. Nevertheless, this transition severely deteriorates the performance due to the large volume change, which is a major concern in the NNM‐based cathodes. According to the quantitative analysis of the in situ XRD patterns (Figure 4c), the interlayer distance shows a huge contraction of ≈22.6% upon transforming from P2 to O2. This inevitably induces large and repetitive lattice strains especially crossing the P2–O2 phase boundaries, leading to mechanical fractures that deteriorate the cyclic stability of the P‐NNM electrode.

**Figure 5:**
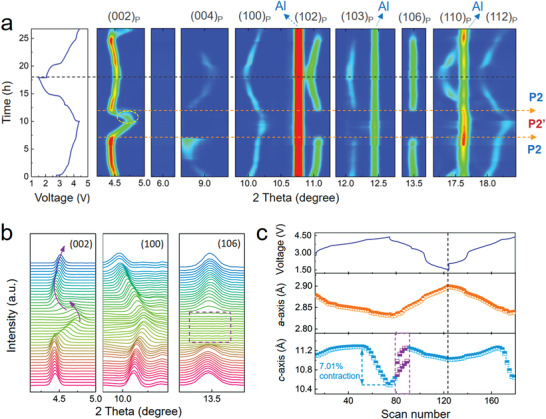
a) Contour plots of the in situ XRD patterns collected from the D‐NNM cell along with the voltage profile. The yellow dashed arrows separate the P2 and P2’ phase regions. b) The typical peak profiles of the in situ XRD patterns. The purple arrows depict the peak evolutions with broken continuity, and the purple dashed square shows the sudden weakening of the peak intensities. c) The lattice evolutions of *a*‐axis (upper) and *c*‐axis (lower) as well as the corresponding voltage profile. The purple dashed lines emphasize the region of structural break associated with the peak splitting.

For the D‐NNM electrode, no newly emerged feature can be observed in the contour plot of in situ the XRD patterns (**Figure** **5**a), indicating that the harmful P2–O2 transition has been suppressed. Different from the frequently observed OP4 phase in Mg‐single‐doped NNM,^[^ ^37^, ^38^, ^39^ ^]^ the Ti/Mg codoped D‐NNM exhibits a reversible solid‐state‐reaction behavior at high voltage. For a more specific view, one typical group of stacking peak profiles are presented in Figure 5b. The (002) peak shifts smoothly to high 2*θ* angle upon deep desodiation, and then reverses back upon initial sodiation. A slight splitting of the (002) peak is also observed at the initial state of discharge. Meanwhile, the (100) peak, corresponding to the *a*‐axis, shows a reversible feature over the entire charging–discharging process. Notably, this successively evolved peak shift also differs from the so‐called “*Z*‐phase” in Fe‐doped NNMs,^[^ ^40^ ^]^ but shares similar characteristics with the solid‐solution H2‐H3 transition in lithium layered oxides (e.g., LiNi*_x_* Mn*_y_* Co*_z_* O_2_ and LiCoO_2_).^[^ ^41^, ^42^, ^43^ ^]^ The former *Z*‐phase is generally identified by a gradual fade of the (002) peak accompanied with the appearance of a new *Z*‐phase feature at a higher 2*θ* angle, which is obviously different from continuously shifted features observed in this study. Furthermore, for both the P‐ and D‐NNM cathodes, the *c*‐oriented reflections, such as (004), (102), and (106) reflection in Figures 4a and 5a, exhibit an abrupt intensity decline at high voltage. This result suggests that the long‐range interlayer ordering is disturbed upon high‐voltage phase transitions.

Figure 5c shows the lattice evolution of the D‐NNM extracted from the in situ XRD patterns based on the typical P2‐type *P*6_3_/*mmc* structural model. At the initial state of charge, the D‐NNM undergoes a contraction along *a*‐axis and an expansion along *c*‐axis, which is almost consistent with the P‐NNM sample. Upon further desodiation, while the *a*‐axis contracts steadily, the length of *c*‐axis quickly decreases at the end of charge. This contraction of interlayer distance, defined as P2–P2’ transition in this work, will give rise to a contraction of 7.01% along *c*‐axis, which is much weaker than the P2–O2 transition in the P‐NNM. The apparent reduction of the interlayer contraction could significantly weaken the repeated lattice strains, hence reducing the electrode cracks for improved cycling stability. Upon further discharging, a slight structural break could be observed in the P2’ phase region, which accords with the splitting of the (002) peak in the XRD patterns (see Figure 5b). This might be derived from the inhomogeneous slab sliding from P2’ to P2 that leads to separated interlayer distances. Notably, although the *P*6_3_/*mmc* structural model could describe the in situ XRD patterns well over the entire charging‐discharging process, it is just a preliminary result in terms of the high‐voltage structure due to the loss of long‐range ordering with overlapped Bragg peaks at high 2*θ* angles. In this regard, a short‐range probe, such as in situ PDF method complementary to the diffraction‐based crystallography,^[^ ^44^, ^45^ ^]^ is deserved to be used to exactly determine the newly emerged P2’ structure in the D‐NNM sample.

**Figure 6:**
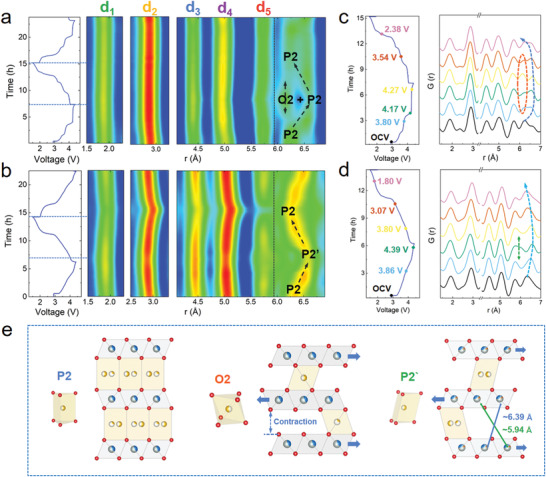
a) G(*r*) contour plots of P‐NNM and b) D‐NNM along with their corresponding voltage profiles. The black arrows show the evolutions of the high‐voltage interlayer distances. c) Stacked profiles of the in situ PDF patterns for P‐NNM and d) D‐NNM at selected voltages. The blue dashed arrows indicate the evolution of interlayer distances at high voltage. The red dashed region in c) shows the appearance of the O2 features in P‐NNM, while the green dashed arrow in d) indicates a newly emerged P2’ feature in D‐NNM at high voltage. e) The schematic diagrams of local structures for P2, O2, and P2’ phases with their Na—O coordinating.

The in situ PDF measurements were performed based on synchrotron X‐ray total scatterings for both P‐NNM and D‐NNM cells. The contour plots of the PDF peak evolutions coupled with their relative voltage profiles are presented in **Figure** **6**a,b, while the corresponding stacked profiles are shown in Figure 6c,d; and Figure S4 (Supporting Information). The first five PDF peaks lying below 6 Å (referred as d1–d5) are related to the in‐plain atom pairs of the octahedral slabs (detailly indicated in Figure S5a, Supporting Information). For both P‐NNM and D‐NNM materials, these in‐plain peaks exhibit very weak shifts during the charging and dis‐charging process, corresponding to the octahedral distortion induced by the TM redox behaviors. On the other hand, the nearest TM interaction between layers lies at ≈5.6 Å in the P2 structure (Figure S5b, Supporting Information), which overlaps with the in‐plain d5 peak and cannot be clearly distinguished. When the *r* distance reaches to above 6 Å, the first interlayer TM interaction starts to be visible in the G(*r*) profiles, which matches the next nearest TM…TM atom pairs at ≈6.3 Å. For the P‐NNM, this interlayer distance continuously expands upon charging, while an additional peak could be observed in the high‐voltage profiles (see Figure 6a,c). Consistent with the in situ XRD results, this newly emerged feature at ≈6.1 Å could be identified as the interlayer TM…TM interaction in the O2 structure. By contrast, for the D‐NNM, the interlayer TM…TM distance shows a reversible evolution without any O2‐derived feature at high voltage. Instead, a weak peak emerging at ≈5.9 Å could be observed when deeply charged (Figure 6b,d). This feature could be an indicator of the P2’ phase in the high‐voltage D‐NNM electrode (elucidated later).

For a more explicit description of the P2’ phase, we refined the in situ PDF patterns within the local‐structure range (< 20 Å). As expected, the P2 structure model (*P*6_3_/*mmc*) fits well to the data of both uncharged P‐NNM and D‐NNM samples (Figure S6a,b, Supporting Information). For the high‐voltage profiles, the P2–O2 two‐phase model is demonstrated appropriate to fit the P‐NNM data (Figure S6c, Supporting Information), which verifies the P2–O2 transition in the charged P‐NNM. During this transition, the coordination environment of Na—O transforms from prismatic to octahedral geometry, accompanied with sliding between layers along (1/3, 2/3, 0) direction (Figure 6e). However, this P2–O2 model fails to describe the high‐voltage D‐NNM data with an agreement factor (*R*_w_) larger than 0.25. Given that the P2’ structure is transformed from P2 through a successive slab sliding, a series of subgroup models are selected from the feasible sliding‐derived structures with symmetrical Na—O geometries, and an orthorhombic *C*222_1_ model gives the best description of the high‐voltage D‐NNM profile (*R*_w_ = 0.156, Figure S6d, Supporting Information). This orthorhombic structure, recognized as the P2’ phase, is transformed from the P2 by sliding slabs along (1/3, 1/3, 0) direction, where the Na^+^ ions between layers reside in diagonal prisms (Figure 6e). From the refinement results, the P2’ structure possesses two distinct interlayer TM…TM interactions, whose distances are 5.94 and 6.39 Å, respectively. This result agrees well with the newly emerged features in the high‐voltage PDF profiles of the D‐NNM sample (Figure 6d). Detailed structural information regarding the high‐voltage O2 and P2’ phases obtained from the PDF profile refinements are given in Tables S5 and S6 (Supporting Information).

**Figure 7:**
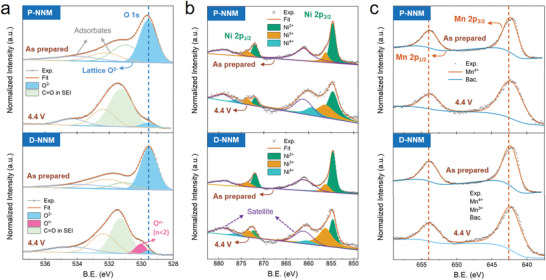
a) The NAP‐XPS peak evolution of a) O 1*s*, b) Ni 2*p*, and c) Mn 2*p* states for the P‐NNM (upper) and D‐NNM (lower) samples. The blue dashed line in a) shows the peak position of lattice O^2−^, while the orange dashed lines in c) indicate the consistent peak positions of Mn^4+^ doublets.

When deeply charged, most of the sodium ions are extracted out of the interlayers, so the stacking of slabs at high voltage are basically governed by O—O electrostatic interaction between layers. Thus, the anomalous P2–P2’ transition in the D‐NNM is expected to be triggered by modified O—O electrostatic force, which is closely related to the oxygen charges shared from the covalent TM—O bonds. With this regard, near‐ambient pressure X‐ray photoelectron spectroscopy (NAP‐XPS) was performed on both uncharged and charged samples to determine the redox chemistries and charge transfer behaviors. Starting from the oxygen (**Figure** **7**a), the peaks with binding energy (BE) at ≈529.5 eV correspond to the lattice O^2−^, whereas the peaks at BEs higher than 531 eV come from the surface oxygen adsorbates.^[^ ^46^ ^]^ When charging to 4.4 V, both P‐NNM and D‐NNM samples exhibit a new peak at ≈531.3 eV, which is typically assigned to the surface deposits from electrolyte decomposition.^[^ ^47^, ^48^ ^]^ Notably, a peak shoulder appears at higher BE side of the lattice O^2−^ (≈530.1 eV) only for the charged D‐NNM sample. This extra component, based on both theories and experiments,^[^ ^49^, ^50^, ^51^, ^52^ ^]^ could arise from the lattice oxidized O*^n^* ^−^ (*n* < 2), while the increase in BE with respect to the lattice O^2−^ can be elucidated by the loss of negative charge on oxygen atoms. As a result, the dopants of Mg and Ti in the D‐NNM lattice could trigger a charge transfer out of oxygen upon deeply charging, so the O—O repulsion between layers is expected to be weakened for modified slab stacking behavior at high voltage.

With the charge outflow of oxygen, there remains an open question as to whether the electrons given away from oxygen in the D‐NNM go to the external circuit for extra capacity, or to the adjacent TMs for charge compensation. To answer this question, the cationic redox behaviors in these two materials were also investigated by XPS. In the typical Ni 2*p* spectra, the spin–orbit doublets at ≈854.7 eV (2*p*3/2) and ≈871.9 eV (2*p*1/2) correspond to the Ni^2+^ state, while the Ni^3+^ state can be distinguished by the splitting holes at ≈856.2 eV (2*p*3/2) and ≈873.4 eV (2*p*1/2), respectively.^[^ ^53^ ^]^ As shown in Figure 7b, the uncharged samples of both P‐NNM and D‐NNM consist largely of Ni^2+^ with a small fraction of Ni^3+^. Upon charging, the Ni^3+^ doublets become stronger accompanied with weakening of the Ni^2+^ peaks. Meanwhile, additional shoulder doublets appear at ≈858.7 and ≈875.9 eV, which are assigned to the Ni^4+^ state. As a result, a multistep Ni^2+^/Ni^3+^/Ni^4+^ redox process is confirmed in both P‐NNM and D‐NNM electrodes. Remarkably, the charged D‐NNM contains less Ni^4+^ but more Ni^3+^ state in comparison with the charged P‐NNM sample. Considering the oxidized O*^n^* ^−^ observed simultaneously, it is suggested that the Ni ions in the charged D‐NNM are reduced by the electrons transferred from the covalently bonded oxygen. In other word, a charge transfer from oxygen to nickel is triggered by codoping Mg and Ti into the D‐NNM lattice, where the electrons from oxygen could partly compensate the charge loss of nickel when deeply charging. On the other hand, the Mn 2*p* spectra shown in Figure 7c can be well assigned to Mn^4+^ state, and no obvious peak shift is observed upon charging for both the materials. Accordingly, the Mn ions in the NNMs are supposed not to participate in the redox reactions. The above XPS results provide key clues of oxygen charge compensation in the D‐NNM. This behavior, as mentioned above, could give rise to a decrease in the interlayer O—O repulsion and therefore play a key role in driving P2–P2’ stacking at high voltage.

**Figure 8:**
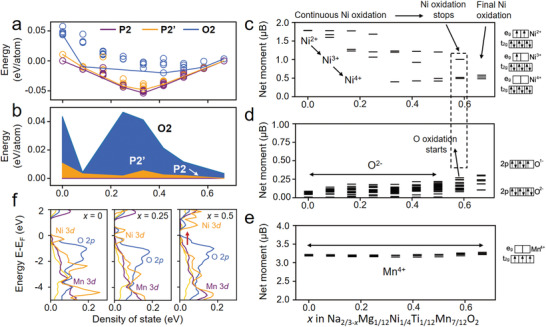
a) Thermodynamic convex hull of the P2, P2’, and O2 phases during the desodiation process. b) Relative energy evolution of the P2’ and O2 phases versus the pristine P2 phase. c) PDOS of the O 2*p* orbitals and Ni/Mn/Ti 3*d* orbitals as a function of Na composition at (left) *x* = 0.00, (middle) *x* = 0.25, and (right) *x* = 0.50 in the D‐NNM.

To understand the underlying mechanism of the as observed phase transition during desodiation, we first compare the formation energies of P2, P2’, and O2 phases of D‐NNM via constructing the corresponding convex hulls upon Na^+^ removal.^[^ ^54^, ^55^ ^]^ Structure models for the P2, P2’, and O2 phases of D‐NNM were created by expanding the primitive cells of these phases and populating the Na and metal sites with Na^+^/vacancy and transition metals (Ni, Mg, Mn, and Ti), then all geometrically different configurations were generated and calculated with the density functional theory (DFT). Figure S7 (Supporting Information) shows the determination of the ground‐state structures with one point stands for the energy of one structure. The final structural models with the lowest total energies are selected as the ground state one. With these structure models as the starting point, the convex hulls were constructed by exploring geometrically distinct Na^+^/vacancy configurations on Na sites of structure models at different compositions (Na^+^/vacancy ratios). For the specific composition, all the Na/vacancy‐ordered configurations were relaxed, and their formation energies were computed according to the following reaction referring to the P2 phase: Na*_x_* MO_2_ → 3/2*x*Na_2/3_MO_2_ (P2) + (1‐3/2*x*)MO_2_ (P2), where M stands for the simulated transition metal composition of Ni_1/4_Mg_1/12_Mn_7/12_Ti_1/12_ (see the Computational Details in the Supporting Information).^[^ ^56^ ^]^ From the calculated desodiation convex hulls of Na_2/3_MO_2_‐MO_2_ shown in **Figure** **8**a, the P2 phase exhibits the lowest formation energy among these three phases at the initial state of charge (0 < *x* < 0.6) while the P2’ phase catches at the end of desodiation (0.6 < *x* < 0.67), indicating a phase transition from P2 to P2’ in the D‐NNM. A similar trend is confirmed by examining the relative energy variations of P2’ and O2 phases versus the P2 phase (Figure 8b). As a comparison, the O2 phase always shows higher‐lying formation energies, implying the absence of the O2 phase upon desodiation of D‐NNM. Using the energetics obtained from the convex hull, we were able to compute the corresponding desodiation voltage profiles of D‐NNM, which well matches the interval measured in the experiments (Figure S8, Supporting Information).

In order to monitor the redox evolutions of TMs and possible participation of O, we examined the oxidation states in the desodiated compositions on the convex hull. The oxidation states can be determined by comparing the calculated magnetizations of TMs and O with the number of unpaired electrons of the corresponding ions with known oxidation states.^[^ ^57^ ^]^ As shown in Figure 8c, continuous Ni oxidation from Ni^2+^ to partially Ni^3+^ and then Ni^4+^ dominates the electrochemical redox process at the early stage of desodiation (0 < *x* < 0.5), accompanied by a decrease in the magnetic moment from around 1.8 *μ*B to 1.1 *μ*B and then to 0.4 *μ*B. Meanwhile, magnetic moments of O and Mn barely vary, indicating the maintenance of their original oxidation states of 2− and 4+, respectively (Figure 8d,e). However, upon further charging, the Ni magnetic moment decrement stops at *x* = 0.5 with the coexistence of Ni^3+^ and Ni^4+^ indicating the halt of capacity contribution from Ni redox (Figure 8c), meanwhile, the O magnetic moments at *x* = 0.5 show noticeable increments (Figure 8d). This implies the participation of O in the high‐voltage redox reactions, validating the oxygen charge compensation as observed in the experiments. In contrast, the Mn ions keep a 4+ charge state during the entire desodiation process (Figure 8e). For further insight, we also checked the projected density of states (pDOS) of TMs (Ni, Mn, Ti) 3*d* and O 2*p* orbitals at several critical desodiation points: *x* = 0, 0.25, and 0.5. At the early stages (*x* = 0 and 0.25), as shown in Figure 8f, the contributions from Ni 3*d* to the valence band immediately below the Fermi level (EF) is larger than that from O 2*p*, so the electrons are preferentially extracted from Ni during the initial state of charge. Upon deep sodiation, surprisingly, the energy state of O 2*p* raises over those of all TMs 3*d* when *x* = 0.5, which confirms the preference of electron given from O instead of TM ions. This result also validates our observations in the magnetic moment examinations. On the other hand, Ti and Mn 3*d* orbitals always show rather low levels throughout the whole desodiation, confirming their inert natures in the redox process.

Altogether, the above results have clarified the driving mechanism of the P2–P2’ transition in the D‐NNM instead of the P2–O2 transition in the P‐NNM. For the typical NNM materials, the P2–O2 transition at high voltage is determined by system total energy. When Na ions are depleted from the interlayers, the O—O electrostatic force becomes critical as it is the major interaction against the layers. So, any change of the O—O repulsion would affect the total energy and thereby the high‐voltage sliding behaviors. Taking the Li‐ion layered oxides as an example, the oxygen charge compensation in this counterpart could result in a decrease of O—O repulsion at high voltage, giving rise to a continuous interlayer contraction that is sustainable for long‐term cycling.^[^ ^27^ ^]^ This behavior is usually referred as H2‐H3 transition or solid‐solution transition. In the present study, we have provided solid evidence that the D‐NNM possesses similar oxygen charge compensation at high voltage. In the same voltage region, the moderate P2–P2’ transition is triggered instead of the typical P2–O2 transition. So, it is natural to suppose that the hindrance of P2–O2 is the result of the oxygen charge compensation as it enables alteration of the systematic total energy by weakening the O—O repulsion.

The above speculation has been further proven by the DFT calculations from combined viewpoints of energetics and electronics. With the gradual extraction of Na^+^, a crossover of formation energies between P2 and P2’ could be observed in the D‐NNM models, which recovers the P2–P2’ transition revealed in the experiments. Meanwhile, the energy of the O2 phase is higher than P2 and P2’ over the entire electrochemical process. So, the P2–O2 transition typically occurred in the P‐NNM is inhibited in the D‐NNM material. Along with the occurrence of P2–P2’ transition, it is found that the energy states of O 2*p* valance electrons raise over those of the TM 3*d* electrons. These shallow‐trapped O 2*p* electrons would compensate the charge loss of TMs through the TM—O covalent bonds, which explains the oxygen charge compensation behavior that triggers the P2–P2’ transition in the D‐NNM. Consequently, the roles of the Ti and Mg dopants can be summarized as the regulators of TM—O covalency and the related O—O repulsion upon charging and discharging, which are the underlying fundamentals of the anomalous P2–P2’ transition in the D‐NNM cathode.

Note that the oxygen charge compensation observed in the D‐NNM should be distinguished from the oxygen redox behavior in the Li‐/Na‐rich systems.^[^ ^4^, ^12^, ^58^ ^]^ In the oxygen redox reaction, the electrons extracted from oxygen will flow to an external circuit and account for extra capacity. By contrast, the XPS results in this study reveal that the appearance of O*^n^* ^−^ (*n* < 2) at high voltage is accompanied by the reduction of Ni ions, so the electrons from the oxygen transfer to Ni ions for charge compensation instead of going to the external circuit for capacity. In addition, the capacity of the D‐NNM is not higher than that of the P‐NNM, so the participation of oxygen in the redox reaction is indistinctive. Furthermore, the charge–discharge profile of D‐NNM shows no obvious high‐voltage plateau of oxygen redox. The sloping characteristic at ≈4.1 V could be related to the newly emerged P2’ phase according to our in situ XRD and in situ PDF results. As a consequence, it is the oxygen charge compensation other than the oxygen redox behavior that is triggered by the Ti and Mg dopants.

## 3 Conclusion

In summary, the P2‐type Ti/Mg codoped NNM layered cathode shows a moderate solid‐solution behavior at high voltage, which differs from the striking P2–O2 phase transition in P‐NNM. This abnormal behavior is driven by the activation of charge compensation through highly hybridized O_2_ *_p_*—TM_3_ *_d_* covalent bonds, as validated by the investigations of geometric and electronic structures as well as first‐principles calculations. In this way, the interlayer O—O electrostatic repulsion is weakened upon deeply charging, which strongly affects the systematic total energy for modulating high‐voltage slab sliding behaviors. Benefiting from the above, the D‐NNM cathode shows a dramatically improved cycling stability compared with the P‐NNM cathode. This study has realized a long‐painted scenario that the layered hosts can store Na^+^ in a similar way to Li^+^ for extended cycle life, opening a new frame‐work for developing high‐performance electrodes for SIBs.

## 4 Experimental Section

Detailed sample synthesis method for pristine and PGE‐NCM811 materials, characterizations, theoretical calculations, and electrochemical measurements are presented in the Supporting Information.

## Conflict of Interest

The authors declare no conflict of interest.

## Supporting information

Supporting Information

## Data Availability

The data that support the findings of this study are available from the corresponding author upon reasonable request.

## References

[1] M. Child , O. Koskinen , L. Linnanen , C. Breyer , Renewable Sustainable Energy Rev. 2018, 91, 321.

[2] N. Nitta , F. Wu , J. T. Lee , G. Yushin , Mater. Today 2015, 18, 252.

[3] H. Zhu , Y. Tang , K. M. Wiaderek , O. J. Borkiewicz , Y. Ren , J. Zhang , J. Ren , L. Fan , C. C. Li , D. Li , X.‐L. Wang , Q. Liu , Nano Lett. 2021, 21, 9997. 34813330 10.1021/acs.nanolett.1c03613

[4] Y. Liu , H. Zhu , H. Zhu , Y. Ren , Y. Zhu , Y. Huang , L. Dai , S. Dou , J. Xu , C.‐J. Sun , X.‐L. Wang , Y. Deng , Q. Yuan , X. Liu , J. Wu , Y. Chen , Q. Liu , Adv. Energy Mater. 2021, 11, 2003479.

[5] D. Kundu , E. Talaie , V. Duffort , L. F. Nazar , Angew. Chem., Int. Ed. 2015, 54, 3431. 10.1002/anie.201410376 25653194

[6] Y. Wang , L. Wang , H. Zhu , J. Chu , Y. Fang , L. Wu , L. Huang , Y. Ren , C.‐J. Sun , Q. Liu , X. Ai , H. Yang , Y. Cao , Adv. Funct. Mater. 2020, 30, 1910327.

[7] N. Yabuuchi , M. Kajiyama , J. Iwatate , H. Nishikawa , S. Hitomi , R. Okuyama , R. Usui , Y. Yamada , S. Komaba , Nat. Mater. 2012, 11, 512. 22543301 10.1038/nmat3309

[8] V. Palomares , P. Serras , I. Villaluenga , K. B. Hueso , J. Carretero‐González , T. Rojo , Energy Environ. Sci. 2012, 5, 5884.

[9] N. Yabuuchi , K. Kubota , M. Dahbi , S. Komaba , Chem. Rev. 2014, 114, 11636. 25390643 10.1021/cr500192f

[10] Y. Li , Y. Lu , C. Zhao , Y.‐S. Hu , M.‐M. Titirici , H. Li , X. Huang , L. Chen , Energy Storage Mater. 2017, 7, 130.

[11] N. Ortiz‐Vitoriano , N. E. Drewett , E. Gonzalo , T. Rojo , Energy Environ. Sci. 2017, 10, 1051.

[12] R. A. House , U. Maitra , M. A. Perez‐Osorio , J. G. Lozano , L. Jin , J. W. Somerville , L. C. Duda , A. Nag , A. Walters , K.‐J. Zhou , M. R. Roberts , P. G. Bruce , Nature 2020, 577, 502. 31816625 10.1038/s41586-019-1854-3

[13] R. J. Clément , P. G. Bruce , C. P. Grey , J. Electrochem. Soc. 2015, 162, A2589.

[14] M. S. Islam , C. A. Fisher , Chem. Soc. Rev. 2014, 43, 185. 24202440 10.1039/c3cs60199d

[15] H. Kim , H. Kim , Z. Ding , M. H. Lee , K. Lim , G. Yoon , K. Kang , Adv. Energy Mater. 2016, 6, 1600943.

[16] M. H. Han , E. Gonzalo , G. Singh , T. Rojo , Energy Environ. Sci. 2015, 8, 81.

[17] P.‐F. Wang , H.‐R. Yao , X.‐Y. Liu , Y.‐X. Yin , J.‐N. Zhang , Y. Wen , X. Yu , L. Gu , Y.‐G. Guo , Sci. Adv. 2018, 4, eaar6018. 29536049 10.1126/sciadv.aar6018 PMC5844706

[18] P. F. Wang , Y. You , Y. X. Yin , Y. S. Wang , L. J. Wan , L. Gu , Y. G. Guo , Angew. Chem., Int. Ed. 2016, 128, 7571.

[19] R. J. Clément , J. Billaud , A. R. Armstrong , G. Singh , T. Rojo , P. G. Bruce , C. P. Grey , Energy Environ. Sci. 2016, 9, 3240.

[20] Y. Liu , X. Fang , A. Zhang , C. Shen , Q. Liu , H. A. Enaya , C. Zhou , Nano Energy 2016, 27, 27.

[21] K. Kaliyappan , J. Liu , B. Xiao , A. Lushington , R. Li , T. K. Sham , X. Sun , Adv. Funct. Mater. 2017, 27, 1701870.

[22] J. H. Jo , J. U. Choi , A. Konarov , H. Yashiro , S. Yuan , L. Shi , Y. K. Sun , S. T. Myung , Adv. Funct. Mater. 2018, 28, 1705968.

[23] D. Yuan , W. He , F. Pei , F. Wu , Y. Wu , J. Qian , Y. Cao , X. Ai , H. Yang , J. Mater. Chem. A 2013, 1, 3895.

[24] J.‐Y. Li , H.‐Y. Lü , X.‐H. Zhang , Y.‐M. Xing , G. Wang , H.‐Y. Guan , X.‐L. Wu , Chem. Eng. J. 2017, 316, 499.

[25] W. Huang , W. Li , L. Wang , H. Zhu , M. Gao , H. Zhao , J. Zhao , X. Shen , X. Wang , Z. Wang , C. Qi , W. Xiao , L. Yao , J. Wang , W. Zhuang , X. Sun , Small 2021, 17, 2104282. 10.1002/smll.202104282 34623019

[26] Y. Huang , H. Zhu , H. Zhu , J. Zhang , Y. Ren , Q. Liu , Nanotechnology 2021, 32, 295701. 10.1088/1361-6528/abf2ff 33780915

[27] O. Kondrakov , H. Geßwein , K. Galdina , L. de Biasi , V. Meded , E. O. Filatova , G. Schumacher , W. Wenzel , P. Hartmann , T. Brezesinski , J. Phys. Chem. C 2017, 121, 24381.

[28] D.‐H. Seo , A. Urban , G. Ceder , Phys. Rev. B 2015, 92, 115118.

[29] C. Delmas , C. Fouassier , P. Hagenmuller , Physica B+C 1980, 99, 81.

[30] E. Talaie , V. Duffort , H. L. Smith , B. Fultz , L. F. Nazar , Energy Environ. Sci. 2015, 8, 2512.

[31] D. H. Lee , J. Xu , Y. S. Meng , Phys. Chem. Chem. Phys. 2013, 15, 3304. 23361584 10.1039/c2cp44467d

[32] Z. Lu , J. Dahn , J. Electrochem. Soc. 2001, 148, A1225.

[33] H. Wang , B. Yang , X.‐Z. Liao , J. Xu , D. Yang , Y.‐S. He , Z.‐F. Ma , Electrochim. Acta 2013, 113, 200.

[34] Y. Wen , B. Wang , G. Zeng , K. Nogita , D. Ye , L. Wang , Chem.– Asian J. 2015, 10, 661. 25641817 10.1002/asia.201403134

[35] M. Medarde , M. Mena , J. Gavilano , E. Pomjakushina , J. Sugiyama , K. Kamazawa , V. Y. Pomjakushin , D. Sheptyakov , B. Batlogg , H. Ott , M. Mansson , F. Juranyi , Phys. Rev. Lett. 2013, 110, 266401. 23848903 10.1103/PhysRevLett.110.266401

[36] Y. Wang , R. Xiao , Y.‐S. Hu , M. Avdeev , L. Chen , Nat. Commun. 2015, 6, 6954. 25907679 10.1038/ncomms7954 PMC4421853

[37] G. Singh , N. Tapia‐Ruiz , J. M. Lopez del Amo , U. Maitra , J. W. Somerville , A. R. Armstrong , J. Martinez de Ilarduya , T. Rojo , P. G. Bruce , Chem. Mater. 2016, 28, 5087.

[38] N. Tapia‐Ruiz , W. M. Dose , N. Sharma , H. Chen , J. Heath , J. W. Somerville , U. Maitra , M. S. Islam , P. G. Bruce , Energy Environ. Sci. 2018, 11, 1470.

[39] S. Kumakura , Y. Tahara , S. Sato , K. Kubota , S. Komaba , Chem. Mater. 2017, 29, 8958.

[40] J. W. Somerville , A. Sobkowiak , N. Tapia‐Ruiz , J. Billaud , J. G. Lozano , R. A. House , L. C. Gallington , T. Ericsson , L. Häggström , M. R. Roberts , U. Maitra , P. G. Bruce , Energy Environ. Sci. 2019, 12, 2223.

[41] L. de Biasi , A. O. Kondrakov , H. Geßwein , T. Brezesinski , P. Hartmann , J. r. Janek , J. Phys. Chem. C 2017, 121, 26163.

[42] S. Zheng , C. Hong , X. Guan , Y. Xiang , X. Liu , G.‐L. Xu , R. Liu , G. Zhong , F. Zheng , Y. Li , X. Zhang , Y. Ren , Z. Chen , K. Amine , Y. Yang , J. Power Sources 2019, 412, 336.

[43] Q. Liu , X. Su , D. Lei , Y. Qin , J. Wen , F. Guo , Y. A. Wu , Y. Rong , R. Kou , X. Xiao , F. Aguesse , J. Bareno , Y. Ren , W. Lu , Y. Li , Nat. Energy 2018, 3, 936.

[44] H. Zhu , Y. Huang , H. Zhu , L. Wang , S. Lan , X. Xia , Q. Liu , Small Methods 2020, 4, 1900223.

[45] H. Zhu , Y. Huang , J. Ren , B. Zhang , Y. Ke , A. K. Y. Jen , Q. Zhang , X.‐L. Wang , Q. Liu , Adv. Sci. 2021, 8, 2003534. 10.1002/advs.202003534 PMC7967088 33747741

[46] J. Alvarado , C. Ma , S. Wang , K. Nguyen , M. Kodur , Y. S. Meng , ACS Appl. Mater. Interfaces 2017, 9, 26518. 28707882 10.1021/acsami.7b05326

[47] D. Foix , M. Sathiya , E. McCalla , J.‐M. Tarascon , D. Gonbeau , J. Phys. Chem. C 2016, 120, 862.

[48] A. Andersson , D. Abraham , R. Haasch , S. MacLaren , J. Liu , K. Amine , J. Electrochem. Soc. 2002, 149, A1358.

[49] G. Assat , D. Foix , C. Delacourt , A. Iadecola , R. Dedryvère , J.‐M. Tarascon , Nat. Commun. 2017, 8, 2219. 29263321 10.1038/s41467-017-02291-9 PMC5738393

[50] Y. Zhang , M. Wu , J. Ma , G. Wei , Y. Ling , R. Zhang , Y. Huang , ACS Cent. Sci. 2020, 6, 232. 32123741 10.1021/acscentsci.9b01166 PMC7047265

[51] J.‐C. Dupin , D. Gonbeau , P. Vinatier , A. Levasseur , Phys. Chem. Chem. Phys. 2000, 2, 1319.

[52] F. Cora , A. Patel , N. M. Harrison , R. Dovesi , C. R. A. Catlow , J. Am. Chem. Soc. 1996, 118, 12174.

[53] K. Hemalatha , M. Jayakumar , P. Bera , A. Prakash , J. Mater. Chem. A 2015, 3, 20908.

[54] A. R. Akbarzadeh , V. Ozoliņš , C. Wolverton , Adv. Mater. 2007, 19, 3233.

[55] B. Wolverton , X.‐Y. Yan , R. Vijayaraghavan , V. Ozoliņš , Acta Mater. 2002, 50, 2187.

[56] Z. Yao , S. Kim , J. He , V. I. Hegde , C. Wolverton , Sci. Adv. 2018, 4, eaao6754. 29795779 10.1126/sciadv.aao6754 PMC5959302

[57] C. Zhan , Z. Yao , J. Lu , L. Ma , V. A. Maroni , L. Li , E. Lee , E. E. Alp , T. Wu , J. Wen , Nat. Energy 2017, 2, 963.

[58] G. Assat , J.‐M. Tarascon , Nat. Energy 2018, 3, 373.

