# Supplementary material for: Unblocking Oxygen Charge Compensation for Stabilized High‐Voltage Structure in P2‐Type Sodium‐Ion Cathode
Source: Adv Sci (Weinh). 2022 Mar 28;9(16):2200498. doi: 10.1002/advs.202200498 (PMC9165493; doi:10.1002/advs.202200498)
Supplement: Supplementary file 1 — Supporting Information [file ADVS-9-2200498-s001.pdf]

ADVANCED SCIENCE
Open Access

Supporting Information

for *Adv. Sci.*, DOI 10.1002/advs.202200498

Unblocking Oxygen Charge Compensation for Stabilized High-Voltage Structure in P2-Type Sodium-Ion Cathode

He Zhu, Zhenpeng Yao, Hekang Zhu, Yalan Huang, Jian Zhang, Cheng Chao Li, Kamila M. Wiaderek, Yang Ren, Cheng-Jun Sun, Hua Zhou, Longlong Fan, Yanan Chen, Hui Xia, Lin Gu*, Si Lan and Qi Liu**

Supporting Information for

Unblocking oxygen charge compensation for stabilized high-voltage P2-type structure in sodium-ion cathode

He Zhu, Zhenpeng Yao, Hekang Zhu, Yalan Huang, Jian Zhang, Cheng Chao Li*, Kamila M. Wiaderek, Yang Ren, Cheng-Jun Sun, Hua Zhou, Longlong Fan, Yanan Chen, Hui Xia, Lin Gu*, Si Lan, Qi Liu*

*Corresponding author. Email: licc@gdut.edu.cn (C.C.L.); l.gu@iphy.ac.cn (L.G.); qiliu63@cityu.edu.hk (Q.L.)

Experimental Section

Synthesis of P-NNM and D-NNM samples. The P-NNM and D-NNM samples used in this study were synthesized *via* a solid-state reaction method. In detail, stoichiometric amounts of $NaCO_3$, NiO, Mn_2O_3, MgO and TiO_2 were firstly ball-milled and mixed for 1 h. Then, the mixed precursors were pressed into pellets under 10 MPa and calcined at 900 ^oC for 15 h in air. The as-prepared samples were cooled to the room temperature naturally and transferred into Ar-protected glove box for further characterizations.

Sample characterization. The SEM and EDS mapping images were taken by JSM-7800F field emission scanning electron microscope (FE-SEM) equipped with an energy-dispersive spectroscopy (EDS) analyzer. The *ex situ* high-energy synchrotron XRD measurements were conducted using 11-ID-C beamline at the Advanced Photon Source (APS) of Argonne National Laboratory (ANL) with the X-ray wavelength of 0.1173 Å. Si (113) single crystal was employed as monochromator for an X-ray beam at 105.7 keV. The Rietveld refinements were performed using Fullprof software[1]. The *ex situ* PDF data was extracted from synchrotron X-ray total scattering by direct Fourier transform of reduced structure function ($F(Q)$, up to Q ~ 26.0 Å) using 11-ID-C beamline at the APS of ANL (λ = 0.1173 Å). PDFgetX3 software was applied to compute the G(r) function[2], and the full-profile refinements of the *ex situ* G(r) patterns were performed using PDFgui package[3]. The structural models were defined in the corresponding unit cells (i.e., $P6_3/mmc$ for P2, $P6_3mc$ for O2 and $C222_1$ for P2') with atomic positions specified in terms of fractional coordinates. The parameters, including lattice constants, atomic position and anisotropic thermal ellipsoids, were allowed to vary until a best fit of the PDF is obtained, using a least-squares approach. The NAP-XPS experiments were performed using a customized, lab-based XPS instrument (Specs Co.) equipped with a monochromic aluminum K_α X-ray source and PHOIBOS 150 HV analyzer.

Electrochemical measurements. The electrochemical tests in this study were carried out using the standard CR2025 coin cells. The positive electrodes were prepared by mixing P- and D-NNM powders with super-P carbon and polyvinylidene fluoride (PVDF) at a weight ratio of 8:1:1 using N-methyl-2-pyrrolidone (NMP) as solvent. The obtained slurry was casted onto an Al foil and dried overnight in a vacuum oven at 90 °C, and then punched into pieces with diameter of 12 mm. The mass loading of active materials of each electrode is about 2-3 mg·cm^{-2}.

All the cells were fabricated in an Ar-filled glove box. The Na metal and glass fiber were adopted as anodes and separators, respectively. $NaClO_4$ in propylene carbonate (PC)/ ethylene carbonate (EC) (1:1 in volume) was employed as electrolyte. The galvanostatic charge/discharge tests was performed on the NEWARE battery system. For the long cyclic stability evaluation, the cells were firstly activated at constant current density of 0.1 C (1 C =170 mA g^{-1}) in the initial three cycles, and evaluated at current density of 1 C in the following cycles.

Collection and analysis of *in situ* synchrotron XRD. The *in situ* synchrotron XRD data were collected at 11-ID-C beamline at the APS of ANL with the X-ray wavelength of 0.1173 Å. The coin cells for the *in situ* XRD measurements were specially designed with holes on both sides of cases sealed with Kapton films as X-ray transparent windows. The fabrication processes of the *in situ* cells were similar to those used in the electrochemical testing. In a typical *in situ* data collection, transmission geometry was applied with a Perkin-Elmer detector to record two-dimensional (2D) diffraction patterns. A standard CeO_2 powder sample was adopted to calibrate sample-to-detector distance, detector tilt angles and the instrumental resolution function. The Fit2D software was used to integrate and calibrate the collected 2D patterns, and the lattice parameters were extracted from the integrated XRD patterns using Fullprof software.

***In situ* PDF measurements.** The *in situ* synchrotron PDF measurements were carried out using 11-ID-C beamline at APS of ANL (~105.7 keV, λ = 0.1173 Å). The electrodes were prepared by mixing P- or D-NNM cathode materials with super-P carbon and PVDF at a weight ratio of 6:2:2, and compressed into pellets. The electrode pellets were then fabricated into a specially designed cell suitable for *in situ* PDF measurements (the AMPIX cell) with a glass fiber as separator, Na metal as anode and $NaClO_4$ in PC/EC (1:1 in volume) as electrolyte[4]. The collection of raw scattering data was similar to that of the *in situ* XRD, except that the sample-to-detector distance is closer for a high value of momentum transfer (Q_{max} ~ 22 Å). The collected 2D scattering images were reduced to one-dimensional data using Fit2D software. The resulted one-dimensional data was corrected with PDFgetX3 software for background and Campton scatterings, and to compute the $G(r)$ functions by Fourier transform. The PDF refinements were carried out using PDFgui software.

First-principles calculations. First-principles density functional theory (DFT) calculations reported in this study were conducted with the Vienna Ab-initio Simulation Package (VASP) (5-

8) with the projector augmented wave (PAW) potentials[9] and the Perdew-Becke-Ernzerhof (PBE) exchange-correlation[10]. A plane wave basis with a cutoff energy of 520 eV and ***Γ***-centered *k*-meshes with a density of 8000 *k*-points per reciprocal atom were used for all calculations. For the structural model, binary Na-$Ni_{1/4}Mg_{1/12}Mn_{7/12}Ti_{1/12}O_2$ ground-state convex hulls were constructed using the structures with the lowest energy for each composition during the desodiation process of $Na_{2/3-x}Ni_{1/4}Mg_{1/12}Mn_{7/12}Ti_{1/12}O_2$ ($0 < x < 2/3$). Then, the stable phases were located by using the convex hull, that is, the set of compounds that have an energy lower than that of any other compound or linear combination of compounds at that composition during the desodiation process. All calculations were spin-polarized, with all transition metal atoms initialized in a high-spin configuration and relaxed to self-consistency. The DFT + U method was used to treat the localized 3*d* electrons of Ni and Mn with a *U* of 6.4 and 3.8[11], obtained by fitting it to experimental and calculated formation enthalpies in a previous study[12].

Voltage profile calculations. The average sodiation/desodiation voltage (relative to Na/Na^+) can be computed using the negative of the reaction free energy per Na added/removed, as shown in *Eq*. (1):

$$V = \frac{\Delta G_{\mathrm{f}}}{F \Delta N_{\mathrm{Na}}} \quad (1)$$

where F is the Faraday constant, ΔN_{Na} is the amount of Na added/removed and ΔG_{f} is the (molar) change in free energy of the reaction[13]. Considering a two-phase reaction between Na_xMO and Na_yMO : $Na_xMO + (y - x)Na \rightarrow Na_yMO$, ΔG_{f} can be approximated by the total internal energies from DFT calculations neglecting the entropic contributions (0 K),

$$\Delta E = E(\mathrm{Na}_y\mathrm{MO}) - E(\mathrm{Na}_x\mathrm{MO}) - (y - x)E(\mathrm{Na}_{\mathrm{metal}}) \quad (2)$$

where $E(\mathrm{Na}_x\mathrm{MO})$ and $\mathrm{E}(\mathrm{Na}_y\mathrm{MO})$ are the DFT energies at the respective compositions. The neglect of entropic contributions means that the sodiation voltage profiles will follow the $T = 0$ K ground state convex hull and consist of a series of constant voltage steps along the two-phase regions of the convex hull, separated by discontinuities which indicate the single-phase compounds on the hull. It is worth mentioning here that sodiation/desodiation do not necessarily proceed through two-phase reactions in practice. Thus, the calculated T = 0 K voltage profiles should be viewed as an approximation to the actual voltage profiles[14]. At finite temperatures (*e.g.*,

room temperature), the voltage drops in the profile become more rounded, due to entropic effects[15].

Supplementary Figures

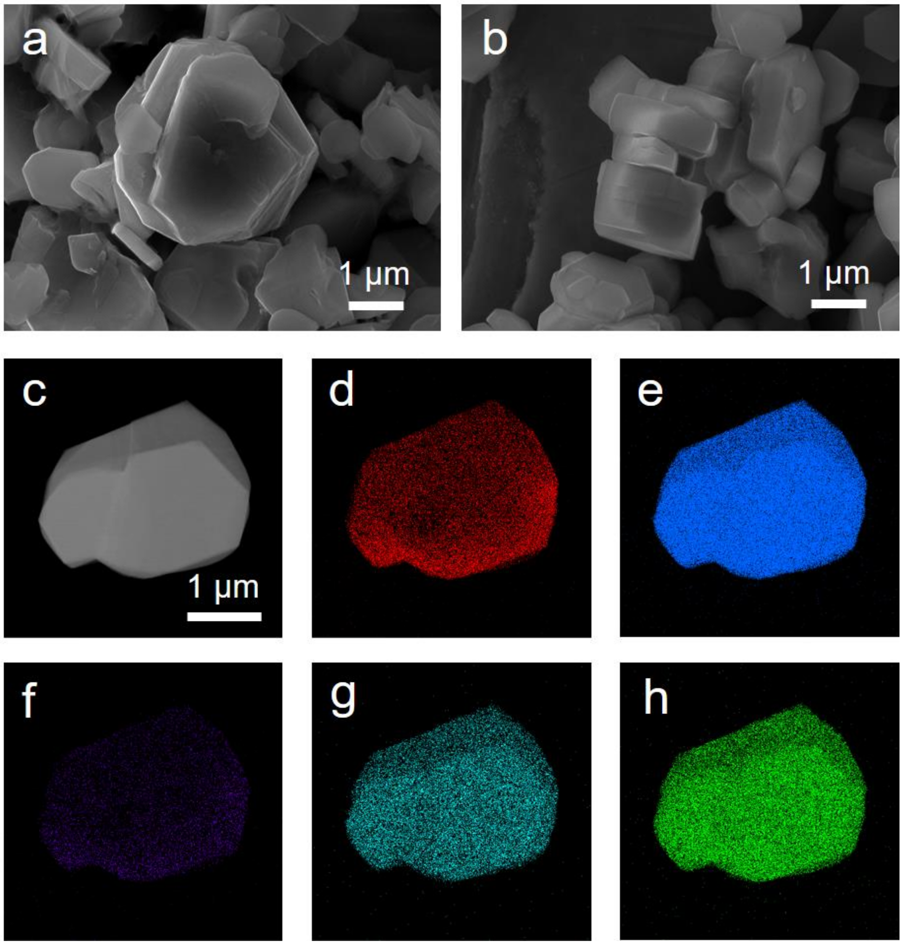

Figure S1. (a) The SEM images of as-prepared P-NNM and (b) D-NNM particles. (c) An individual D-NNM particle along with the EDS mapping images for (d) Na, (e) Mn, (f) Mg, (g) Ti and (h) Ni.

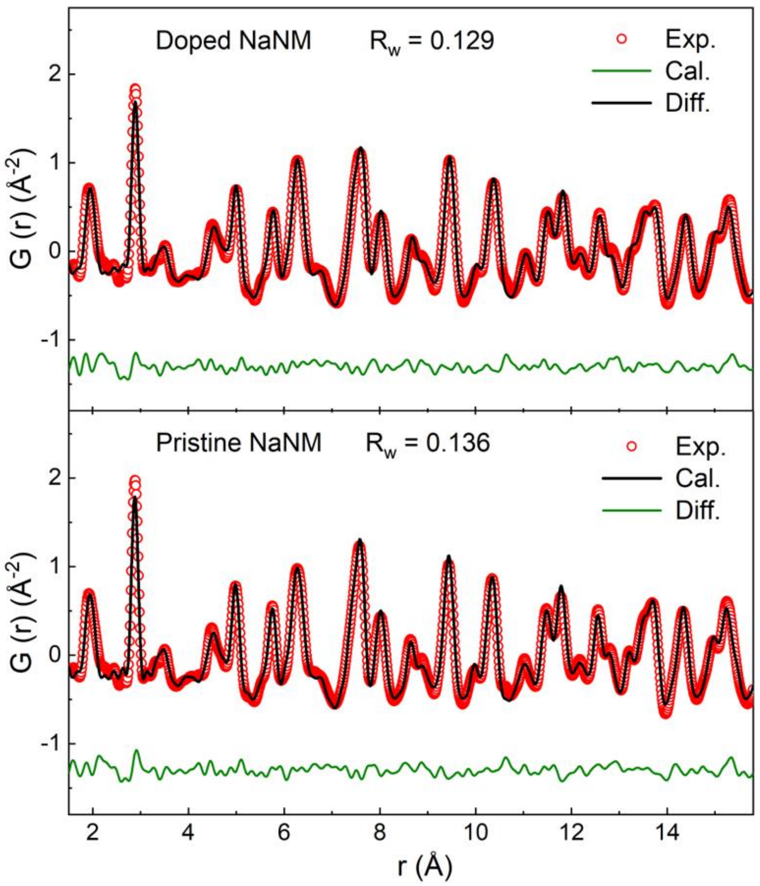

Figure S2. PDF refinement patterns for D-NNM (upper) and P-NNM (bottom) materials with the hexagonal $P6_3/mmc$ model.

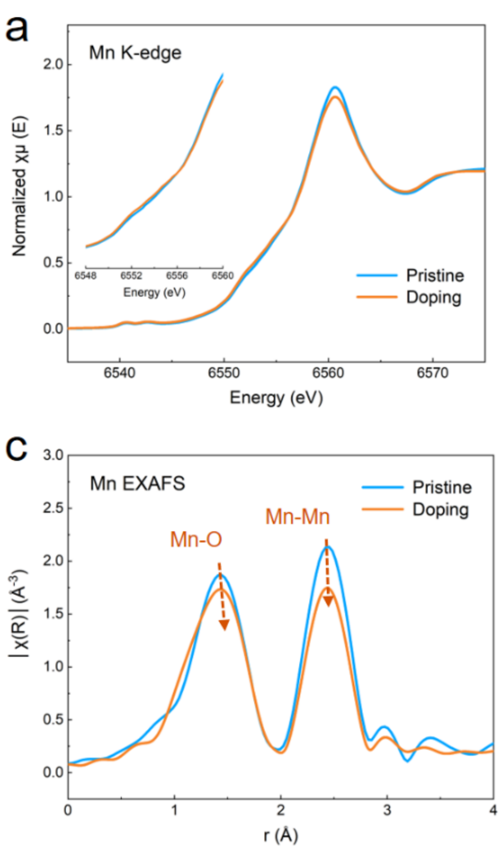

Figure S3. (a) The XANES spectra of Mn K-edge and (b) Ni K-edge for both P- and D-NNM materials. The insets enlarge the shifts of absorption edges. (c) The EXAFS of Mn K-edge and (d) Ni K-edge for P- and D-NNM materials. The arrows indicate the peak shifts of the first and second coordination shells.

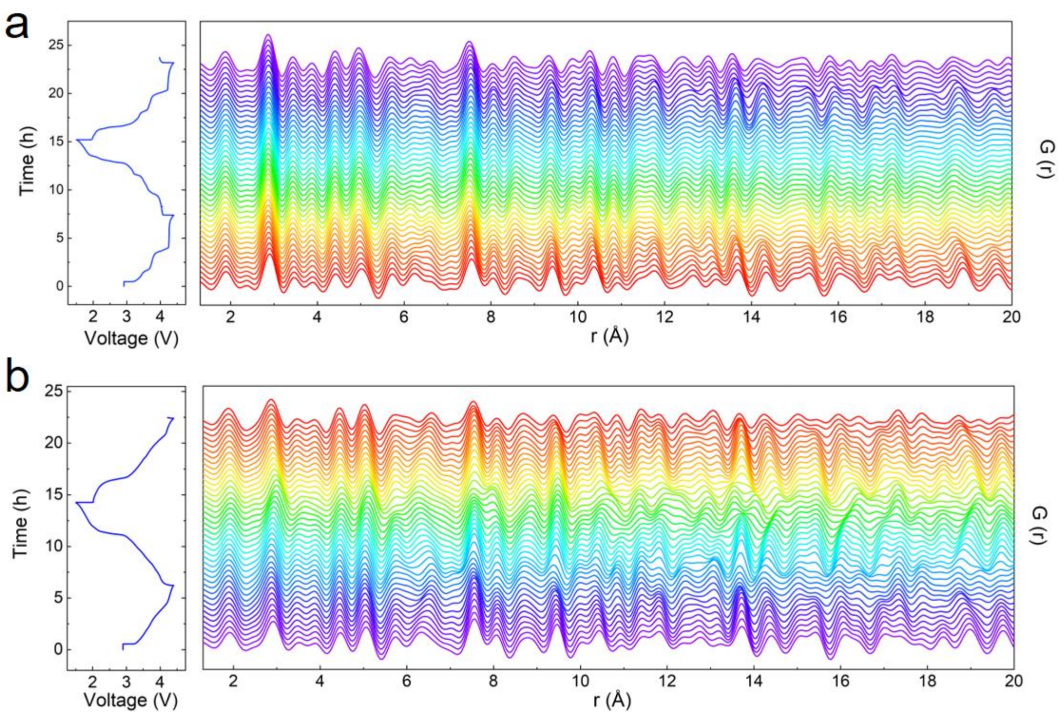

Figure S4. Contour plots *in situ* PDF patterns for (a) P-NNM and (b) D-NNM materials coupled with their corresponding voltage profiles.

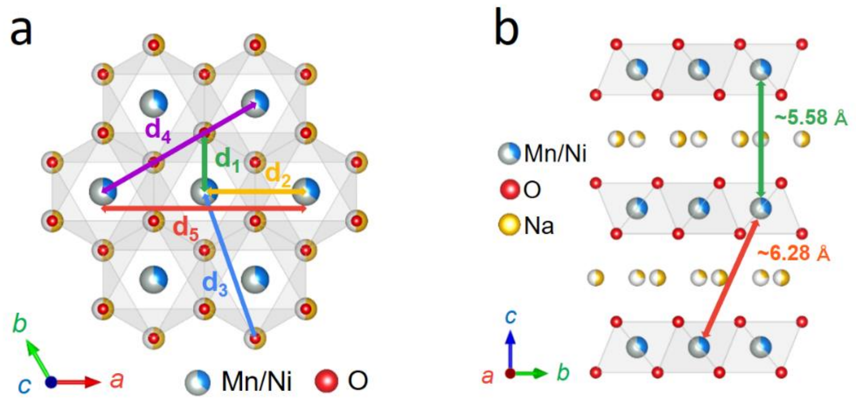

Figure S5. (a) The in-plain atom pairs viewed along *c* direction. The labels from d_1 to d_5 correspond to the first five peaks in the PDF G(*r*) patterns. (b) The interlayer atomic distances viewed along *a*/*b* direction. The green and red arrows indicate the nearest and next nearest TM interactions between layers.

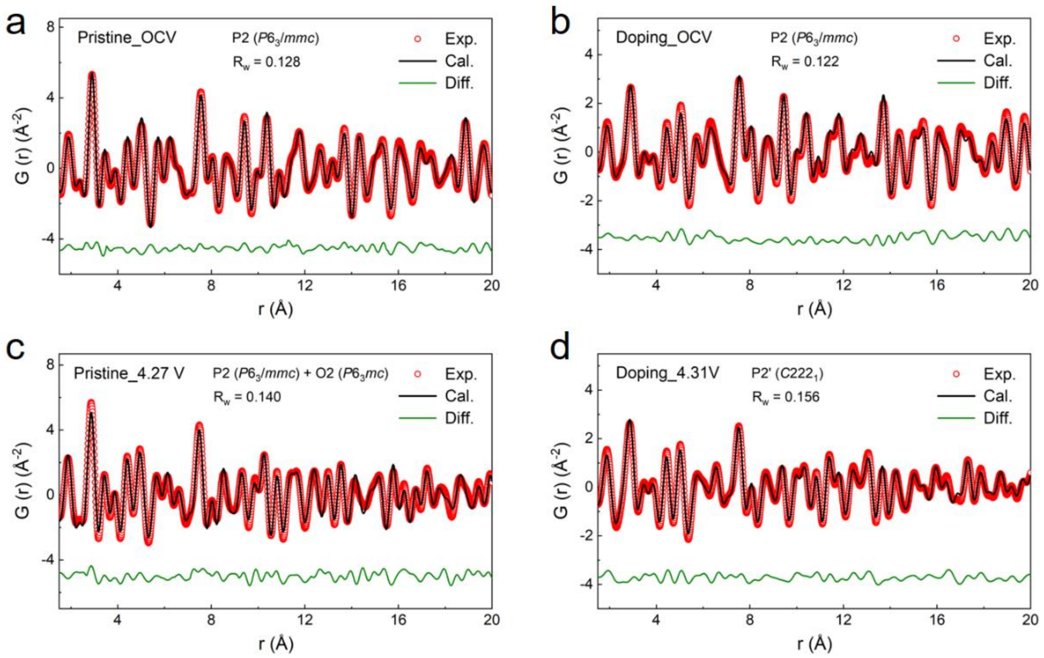

Figure S6. (a) Full profile refinement patterns of P-NNM and (b) D-NNM at open circuit voltage (OCV) adopting P2-type $P6_3/mmc$ structural models. (c) The refinement pattern of 4.27 V-charged P-NNM with P2-O2 two-phase model. (d) The refinement pattern of 4.31 V-charged D-NNM with P2' structural model.

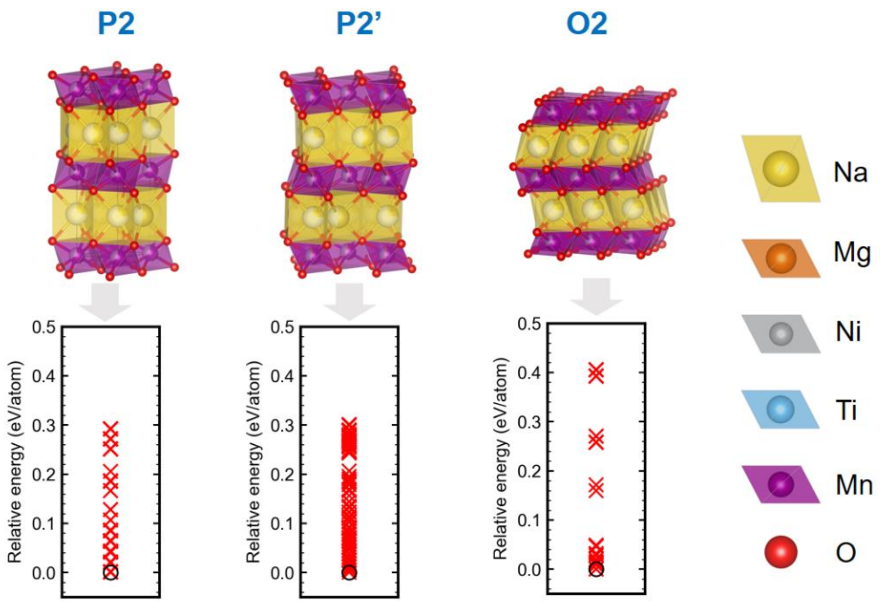

Figure S7. The final structural models of P2 (left), P2' (middle) and O2 (right) with lowest total energies.

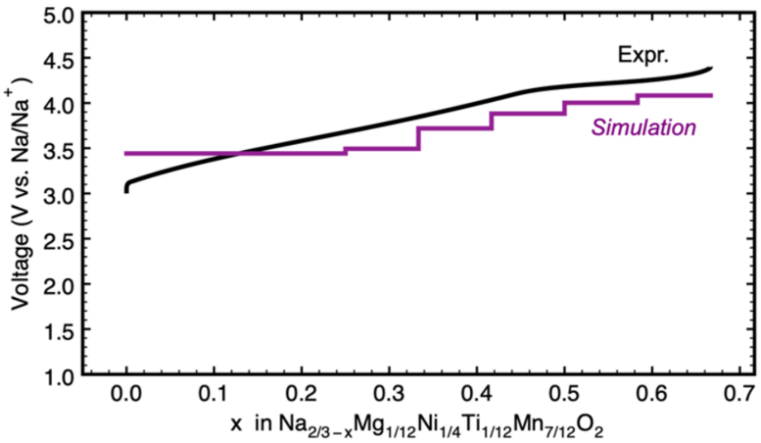

Figure S8. Comparison of experimental and simulated voltage profiles for D-NNM cathode.

Supplementary Tables

Table S1. The unit cell parameters of P-NNM extracted from XRD Rietveld refinement.

Atom	X	Y	Z	Occ.
**Na1**	0	0	0.25	0.232(4)
**Na2**	0.67	0.33	0.25	0.461(6)
**Ni**	0	0	0	0.333
**Mn**	0	0	0	0.667
**O**	0.333	0.667	0.0877(2)	1
a = 2.88554(3) Å, c = 11.1791(3) Å, V = 80.610(2) Å^3				

Table S2. The unit cell parameters of D-NNM extracted from XRD Rietveld refinement.

Atom	X	Y	Z	Occ.
**Na1**	0	0	0.25	0.252(3)
**Na2**	0.67	0.33	0.25	0.445(6)
**Ni**	0	0	0	0.25
**Mn**	0	0	0	0.55
**O**	0.333	0.667	0.0877(2)	1
**Mg**	0	0	0	0.083
**Ti**	0	0	0	0.117
a = 2.89809(3) Å, c = 11.1862(3) Å, V = 81.365(2) Å^3				

Table S3. The unit cell parameters of P-NNM extracted from PDF refinement.

Atom	X	Y	Z	Occ.
**Na1**	0	0	0.25	0.225 (2)
**Na2**	0.67	0.33	0.25	0.457(3)
**Ni**	0	0	0	0.333
**Mn**	0	0	0	0.667
**O**	0.333	0.667	0.0912(2)	1
a = 2.8812(1) Å, c = 11.160(2) Å, V = 80.23(2) Å^3				

Table S4. The unit cell parameters of P-NNM extracted from PDF refinement.

Atom	X	Y	Z	Occ.
**Na1**	0	0	0.25	0.241(3)
**Na2**	0.67	0.33	0.25	0.452(3)
**Ni**	0	0	0	0.25
**Mn**	0	0	0	0.55
**O**	0.333	0.667	0.0913(4)	1
**Mg**	0	0	0	0.083
**Ti**	0	0	0	0.117
a = 2.8930(2) Å, c = 11.160(3) Å, V = 81.89(2) Å^3				

Table S5. The unit cell parameters of the O2 phase (space group: $P6_3mc$) in high-voltage P-NNM extracted from *in situ* PDF refinement.

Atom	X	Y	Z	Occ.
Na	0.333	0.667	0.25	0.192 (5)
Ni	0.333	0.667	0	0.333
Mn	0	0	0	0.667
O	0.667	0.333	0.0914(4)	1
a = 2.881 (3) Å, c = 8.807(3) Å, V = 63.31(5) Å^3				

Table S6. The unit cell parameters of the P2' phase (space group: $C222_1$) in high-voltage D-NNM extracted from *in situ* PDF refinement.

Atom	X	Y	Z	Occ.
Na1	0	0	0	0.06(5)
Na2	0.67	0.33	0.25	0.10(6)
Ni	0	0.167	0.75	0.25
Mn	0	0.167	0.75	0.55
O	0.167	0.333	0.159(5)	1
Mg	0	0.167	0.75	0.083
Ti	0	0.167	0.75	0.117
a = 4.990(2) Å, b = 2.881(2) Å, c = 10.742(4) Å, V = 154.46(3) Å^3				

Reference

[1] J. Rodríguez-Carvajal, Recent advances in magnetic structure determination by neutron powder diffraction. *Physica B Conden. Matter* **192**, 55-69 (1993).

[2] P. Juhás, T. Davis, C. L. Farrow, S. J. Billinge, PDFgetX3: a rapid and highly automatable program for processing powder diffraction data into total scattering pair distribution functions. *J. Appl. Crystallogr.* **46**, 560-566 (2013).

[3] C. Farrow, P. Juhas, J. Liu, D. Bryndin, E. Božin, J. Bloch, T. Proffen, S. Billinge, PDFfit2 and PDFgui: computer programs for studying nanostructure in crystals. *J. Phys. Conden. Matter* **19**, 335219 (2007).

[4] O. J. Borkiewicz, B. Shyam, K. M. Wiaderek, C. Kurtz, P. J. Chupas, K. W. Chapman, The AMPIX electrochemical cell: a versatile apparatus for *in situ* X-ray scattering and spectroscopic measurements. *J. Appl. Crystallogr.* **45**, 1261-1269 (2012).

[5] G. Kresse, J. Hafner, *Ab initio* molecular dynamics for liquid metals. *Phys. Rev. B* **47**, 558 (1993).

[6] G. Kresse, J. Hafner, *Ab initio* molecular-dynamics simulation of the liquid-metal-amorphous-semiconductor transition in germanium. *Phys. Rev. B* **49**, 14251 (1994).

[7] G. Kresse, J. Furthmüller, Efficiency of *ab-initio* total energy calculations for metals and semiconductors using a plane-wave basis set. *Comput. Mater. Sci.* **6**, 15-50 (1996).

[8] G. Kresse, J. Furthmüller, Efficient iterative schemes for *ab initio* total-energy calculations using a plane-wave basis set. *Phys. Rev. B* **54**, 11169 (1996).

[9] P. E. Blöchl, Projector augmented-wave method. *Phys. Rev. B* **50**, 17953 (1994).

[10] J. P. Perdew, M. Ernzerhof, K. Burke, Rationale for mixing exact exchange with density functional approximations. *J. Chem. Phys.* **105**, 9982-9985 (1996).

[11] S. Dudarev, G. Botton, S. Savrasov, C. Humphreys, A. Sutton, Electron-energy-loss spectra and the structural stability of nickel oxide: An LSDA+U study. *Phys. Rev. B* **57**, 1505 (1998).

[12] L. Wang, T. Maxisch, G. Ceder, Oxidation energies of transition metal oxides within the GGA+*U* framework. *Phys. Rev. B* **73**, 195107 (2006).

[13] M. Aydinol, A. Kohan, G. Ceder, K. Cho, J. Joannopoulos, *Ab initio* study of lithium intercalation in metal oxides and metal dichalcogenides. *Phys. Rev. B* **56**, 1354 (1997).

[14] M. K. Chan, C. Wolverton, J. P. Greeley, First principles simulations of the electrochemical

lithiation and delithiation of faceted crystalline silicon. *J. Am. Chem. Soc.* **134**, 14362-14374 (2012).

[15] C. Wolverton, A. Zunger, First-principles prediction of vacancy order-disorder and intercalation battery voltages in Li_xCoO_2. *Phys. Rev. Lett.* **81**, 606 (1998).
